# Effects of copal resin extraction on the diversity and composition of species in tropical deciduous forests

**DOI:** 10.1038/s41598-023-31423-z

**Published:** 2023-03-14

**Authors:** Rosa María García-Núñez, Julio César Buendía-Espinoza, Selene del Carmen Arrazate-Jiménez, Elisa del Carmen Martínez-Ochoa

**Affiliations:** 1 Maestría en Ciencias en Agroforestería para el Desarrollo Sostenible, Departamento de Suelos, Universidad Autónoma Chapingo, Km 38.5 Carretera México-Texcoco, 56230 Texcoco, Estado de México Mexico; 2 Área de Agronomía, Departamento de Preparatoria Agrícola, Universidad Autónoma Chapingo, Km 38.5 Carretera México-Texcoco, 56230 Texcoco, Estado de México Mexico

**Keywords:** Ecology, Behavioural ecology, Biodiversity, Community ecology, Ecological modelling

## Abstract

Composition and floristic diversity of ecosystems subject to overexploitation, such as tropical deciduous forests where copal resin (*Bursera bipinnata, Bursera copallifera*) is extracted, are of great importance for understanding the ecological functioning of these ecosystems. This study analyzed the species composition and diversity in a natural population subject to copal extraction in San Juan Raboso Izúcar de Matamoros, Puebla, Mexico. A total of 54 sampling units were established, and the number of individuals and crown diameter for each tree species were recorded. For shrubs, succulents, acaulescent rosetophytes and climbers, the number of individuals and the area of cover were quantified. Based on the parameters of abundance, frequency, and relative dominance, the importance value index (IVI) was calculated. Diversity was evaluated using the Shannon index $(H {^{\prime}})$. In total, 29 species were identified, distributed across 11 botanical families and 21 genera. The Fabaceae family was the richest, followed by the Burseraceae family, which includes the species that extract copal, but *Opuntia streptacantha* was the species with the most ecological weight. In this study, the Shannon index $({H}^{^{\prime}})$ averaged 1.45, which indicates that the community was mildly diverse.

## Introduction

Tropical deciduous forests are home to many plant species, influence the climate, regulate river flow, and provide timber as well as non-timber forest products, such as tannins, fruits, fibers, palms, resins, and so on^1,2^. In Mexico, tropical deciduous forests have been severely impacted by the expansion of the agricultural frontier, population growth, and the search for better living conditions^3,4^, affecting biodiversity and compromising ecological balance^5^.

The Balsas depression exhibits one of the maximum expressions of this vegetation since it possesses a great floristic richness^6^, in part because it has a great diversity of climatic conditions and relationships with neighboring provinces^7^. Due to its diversity and endemism, it is one of the areas of Mexico with the greatest botanical interest, but it has been little researched^8,9^. In Puebla, for instance, the remnants of tropical deciduous forests located in the upper Balsas watershed provide a wide range of non-timber forest products such as fruit, aromatic resins, essential oils, tannins and handicrafts, as well as firewood and timber used to make rustic furniture^7,10^. Several of these species have been used as sustainable economic alternatives in dry hot climates^11^, such as the species of Burseraceae family, particularly the genus *Bursera*, in Izúcar de Matamoros, Puebla, Mexico^6^, which are used to produce aromatic resins, such as copal^12^. Their overexploitation can result in the death of trees, extinction of species, and the loss of genetic diversity^13^, as there is no planning for their exploitation, nor is there an evaluation of their ecological impact.

Tropical deciduous forests are damaged by inadequate copal harvesting. As a result of inefficient harvesting methods, Abad-Fitz et al.^14^ documented a low density of copal species in unmanaged extraction areas in the state of Morelos, Mexico. Similarly, in another study on copal abundance, carried out in Los Sauces and Pitzotlán, in Tepalcingo, Morelos, Mexico, important communities for copal resin harvesting, a significant change in vegetation was identified in the community of Pitzotlán; even though the area studied was 127.5 ha, 8.5 ha larger than Los Sauces, only 96 trees were harvested, thus 167 trees less than Los Sauces. In comparing the communities, it is evident that in Pitzotlán there has been a change in the vegetation structure as a result of human activity, where copal trees have declined and renewal of trees has been limited due to overgrazing^15^.

Identifying the composition and floristic diversity of ecosystems subject to overexploitation is essential to understanding their ecological functioning, to improving the management of harvested species, and to assisting in the development of conservation plans and sustainable use of natural resources^16^. In San Juan Raboso, Izúcar de Matamoros, tropical deciduous forest is suffering from overexploitation. This is due to an increase in copal cultivators, who, owing to the increasing demand for copal resin, promote an inadequate extraction process and severely damage the trees, causing a decrease in their natural populations^12^. The study, quantification, and analysis of these forests are important for understanding the natural world and the effects of human activities^16,17^.

This study aimed to evaluate the composition and diversity of species present in a natural population of tropical deciduous forest that has been subjected to copal harvesting in order to identify their spatial patterns as well as abundance to aid in their management and conservation.

## Materials and methods

### Study area

**Figure 1:**
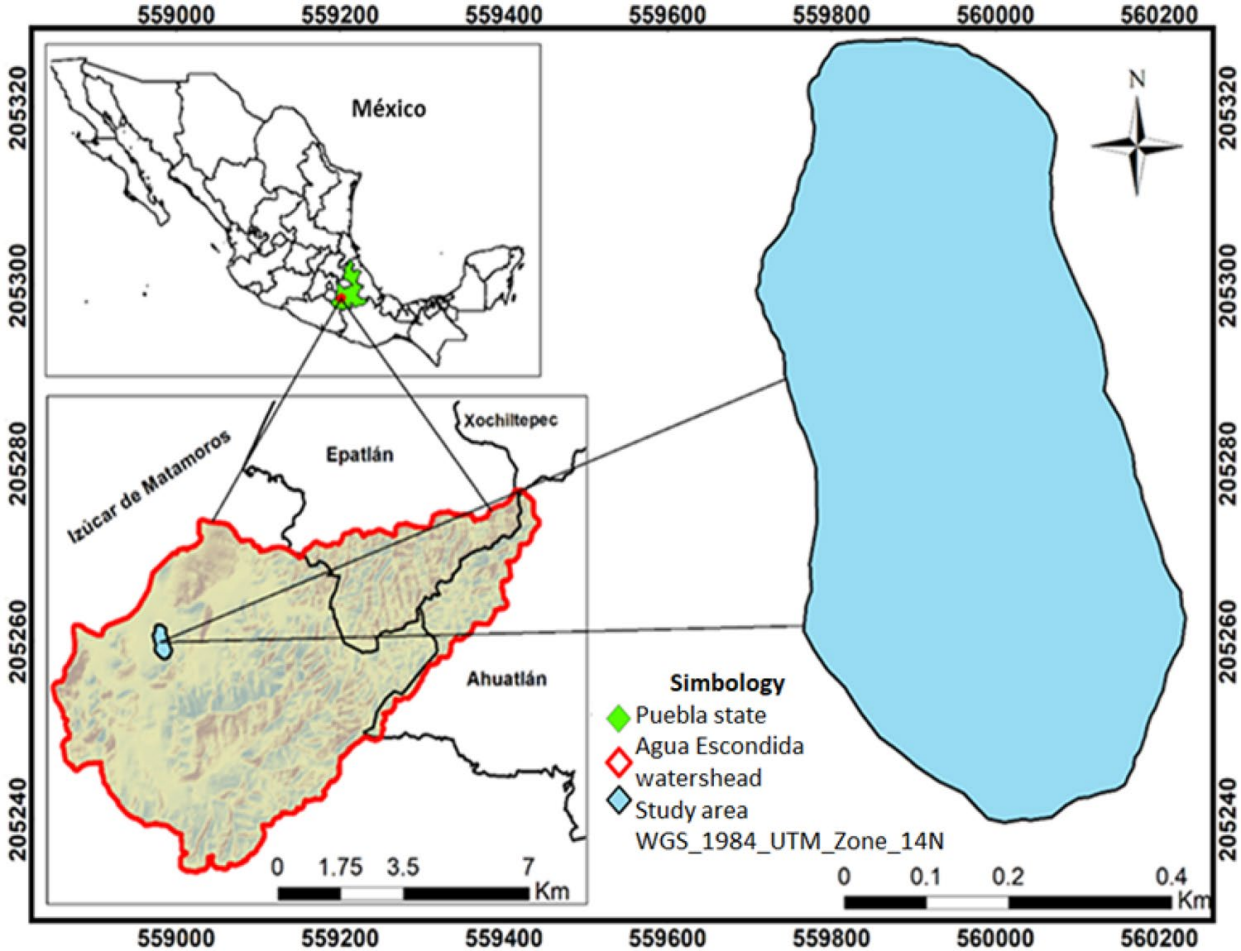
Area of study and location of the experimental site within the Agua Escondida watershed, Puebla Mexico (Buendía-Espinoza JC, et al. 2022, available at https://doi.org/10.3390/su14138047)^20^.

The study area was the Agua Escondida watershed located in the municipalities of Izúcar de Matamoros, Epatlán, Ahuatlán and Xochiltepec, in the Puebla state, with elevations from 1100 to 1 600 m and an area of 6492 ha. With a temperature range of 19–25 °C and a precipitation range of 700–900 mm per year. The predominant soils are leptosol, vertisol and regosol. There are two main climates within the watershed: warm sub-humid with summer rains and semi-warm sub-humid with summer rains. Primary land use is agriculture, and the predominant vegetation type is tropical deciduous forest^17–20^. The experimental site was in the Ejido de San Juan Raboso, municipality of Izúcar de Matamoros, located in the region of the Cerro de la Virgen, within the watershed (Fig. 1), with an elevation 1 282 m and an area of 30.8 ha. Tropical deciduous forest is predominant in this area, typical of an area with two distinct seasons of moisture availability, rainy and dry^7^, as well as species that shed their leaves during the dry season and a variety of species with scaly bark that is similar to papyrus, or with thorny or corky protuberances, with sparse crowns^20,21^. The experimental site is for communal use. So, its resources belong to everyone and nobody at the same time, and the copal trees are some of its most exploited resources. This is due to the constant demand for resin due to their religious beliefs, incorporating it in various celebrations of different ethnic groups^15^, mainly in the fall. The copal resin extraction season takes place during three months, from August to October^14^. The copaleros begin with the marking, which refers to the selection of the best trees and make the first incision. After that, they have to wait an average of one week for the resin to begin to emanate. More incisions are made as many times as necessary until the end of the harvesting season^15^. In addition to this, the area is located less than 5 km from the community of San Juan Raboso, which makes it very easy to access. Other factors impact the natural area such as overgrazing, scarce natural regeneration of copal, and the lack of an adequate resin extraction method among others.

### Vegetation sampling

**Figure 2:**
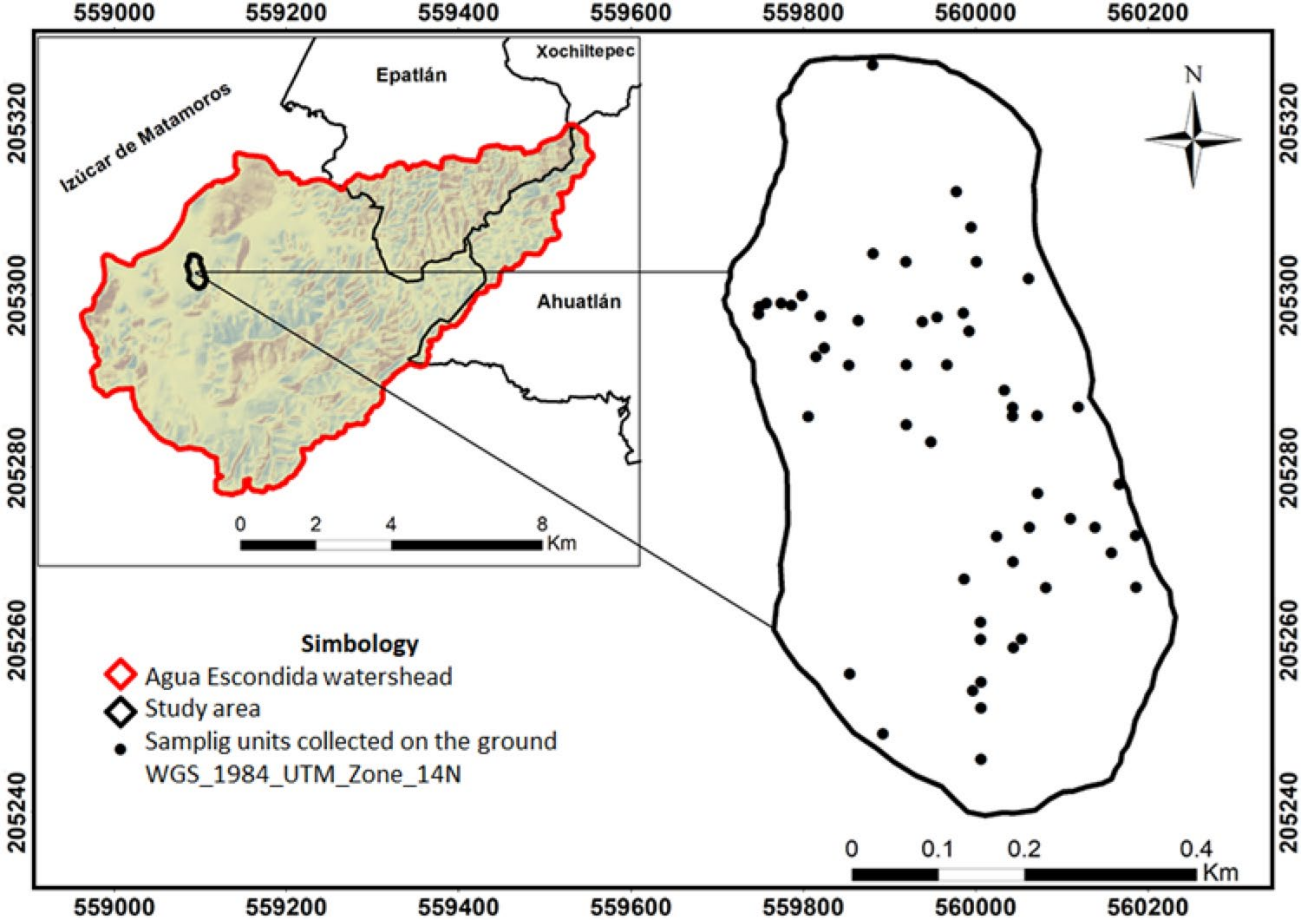
Location of the sampling units in the study area (Elaborated with ArcGIS 10.8, https://pro.arcgis.com/es/pro-app/latest/get-started/download-arcgis-pro.htm).

The data used in the study area was collected using simple random sampling, and the geographic coordinates of each sample unit were obtained and mapped. The sample size was 54 sampling units, based on a 100 m^2^ quadrat^22^, which are indicated with black squares on the map (Fig. 2). Species found in each sampling unit were recorded and taxonomically identified. The common names of the plants and information regarding some of their uses have been provided by local residents. For each tree species, the variables measured were number of individuals and crown diameter, calculated by averaging two crosswise measurements with a tape measure, while for shrub species, succulents, and acaulescent rosettes, the variables measured were the number of individuals and canopy area^23^.

### Importance value index

The Importance Value Index, IVI, indicates the ecological importance of a given species within a plant community, and is based on the sum of the structural parameters of abundance, frequency, and relative dominance^24,25^.

1 $$IVI={AR}_{i}+{DR}_{i}+{FR}_{i},$$

where ${AR}_{i}$ represents the relative abundance, ${DR}_{i}$ the relative dominance, and ${FR}_{i}$ the relative frequency.

#### Absolute and relative abundance

The relative and absolute abundance of the species was calculated as follows:

2 $${A}_{i}=\left(\frac{{N}_{i}}{S}\right),$$

3 $${AR}_{i}=\left(\frac{{A}_{i}}{\sum_{i=1}^{n}{A}_{i}}\right)\times 100,$$

where ${A}_{i}$ represents the absolute abundance, ${\mathrm{AR}}_{\mathrm{i}}$ represents the relative abundance of species $i$ relative to the total abundance, ${N}_{i}$ represents the number of individuals of species $i$, and $S$ represents the sampling area (ha).

#### Absolute and relative frequency

The absolute and relative frequency were calculated using the following formula:

4 $${F}_{i}=\left(\frac{{P}_{i}}{NS}\right),$$

5 $${FR}_{i}=\left(\frac{{F}_{i}}{\sum_{i=1}^{n}{F}_{i}}\right)\times 100,$$

where ${F}_{i}$ is the absolute frequency, ${FR}_{i}$ is the relative frequency of species $i$ when compared to the total frequency, ${P}_{i}$ is the number of sampling sites where species $i$ is present, and $NS$ is the total number of sampling sites.

#### Absolute and relative dominance

The relative and absolute dominance of the species in the different sampling units was estimated as follows:

6 $${D}_{i}=\left(\frac{{Ab}_{i}}{S(ha)}\right),$$

7 $${DR}_{i}=\left(\frac{{D}_{i}}{\sum_{i=1}^{n}{D}_{i}}\right)\times 100,$$

where ${D}_{i}$ is the absolute dominance, ${DR}_{i}$ is the relative dominance of species $i$ with respect to total dominance, ${Ab}_{i}$ is the canopy area of species $i$ and $S$ the land area (ha).

The abundance, frequency, and dominance values were determined according to Mora-Donjuán et al.^26^.

### Measuring species diversity

Diversity of species is an indicator of the character and structure of a community when compared with another.

#### Shannon index

This index measures diversity based on the number of species and their abundance^27^. Shannon's index is conceptually a measure of the level of uncertainty associated with a random selection of an individual within a given community. It is widely used to measure changes in diversity resulting from changes in environmental factors, and is, therefore, considered a good indicator of the reliability of the estimated value^28,29^.

8 $$H^{\prime}=-\sum_{i=1}^{S}{P}_{i}\times \mathit{ln}\left({P}_{i}\right),$$

9 $${P}_{i}=\frac{{n}_{i}}{N},$$

where $S$ represents the number of species present, $N$ represents the number of individuals, and ${n}_{i}$ represents the number of individuals of species $i$.

$H^{\prime}$ is limited between zero and $lnS$ and tends to zero in communities with low diversity, whereas it is equal to the logarithm of specific richness in communities with maximum equitability. Species richness is defined as the number of species present in a community^30^. Shannon values between 0 and 1.35 indicate low diversity, 1.36–3.5 indicate medium diversity, and more than 3.5 indicates high diversity^31^.

#### Bootstrap confidence interval, BCI

The Confidence Interval of the Shannon index was constructed using the Bootstrap Confidence Interval method. The bootstrap estimator ${H}^{*}$ follows an approximately normal distribution with mean $H$ and standard deviation ${\sigma }_{H}$; accordingly, there is a $(1-\alpha )$ probability that:

10 $$\left(H+{z}_{\alpha/2}{\sigma }_{H}<{H}^{*}<H+{z}_{\left(1-\alpha/2\right)}{\sigma }_{H}\right),$$

for any random sample used to calculate ${H}^{*}$^32^. The confidence interval, then, is:

11 $$\left( {\bar{H}^{*} + z_{{\alpha /2}} \sigma _{H}^{*} < H < \bar{H}^{*} + z_{{\left( {1 - \alpha /2} \right)}} \sigma _{H}^{*} } \right),$$

where ${\overline{H} }^{*}$ is the estimated mean of the bootstrap parameter and ${\sigma }_{H}^{*2}$ is its variance.

12 $${\overline{H} }^{*}=\frac{1}{B}{\sum }_{j=1}^{B}{H}_{j}^{*} \; and \; {\sigma }_{H}^{*2}=\frac{1}{B}{\sum }_{j=1}^{B}{\left({H}_{j}^{*}-{\overline{H} }^{*}\right)}^{2},$$

where B is the number of bootstrap samples taken. Bootstrap interval coverage was calculated with a nominal probability of 0.95 and 1000 replicates^33,34^.

#### Shannon index interpolation

Spatial interpolation is a mathematical procedure used to estimate the value of an attribute, in this case, the Shannon diversity index, at a particular location or cell based on measurements taken at different points. Interpolating the Shannon index spatially was achieved by transforming the point results from the sampling points into a continuous space, so that their spatial pattern could be compared to the observations of the point sources^35^. The diversity index was interpolated using the r software package interp^36^.

Statistical analyses were performed using R version 3.6.3^37^.

## Results

### Floristic composition

A total of 1793 ind ha^−1^ was found represented in 29 species belonging to 11 botanical families and 21 genera (Table 1). A total of thirteen tree species, nine shrub species, four succulents listed in Nom-059-Semarnat-2010^38^ under the special protection category, two acaulescent rosetophytes and one climbing species were found. According to Bravo et al.^39^, the woody climbing species *Marsdenia zimapanica* should be considered in the shrub stratum due to the thickness of its stem.

**Table 1:** List of common, scientific, and family names of the species found in the study area (arranged alphabetically by family).

Scientific name	Common name	Family	Life form
*Cyrtocarpa procera* Kunth	Coco de cerro	Anacardiaceae	Tree
*Marsdenia zimapanica* Hemsl	Tecuampatli	Apocynaceae	Climber
*Agave ghiesbreghtii* Lem. ex Jacobi	Maguey	Asparagaceae	Acasucculentes rhosophytes
*Agave karwinskii* Zucc	Maguey oscuro	Asparagaceae	Acasucculentes rhosophytes
*Ceiba aesculifolia* (Kunth) Britten & Baker f	Pochote	Bombacaceae	Tree
*Bursera aloexylon* (Schiede ex Schltdl.) Engl	Linaloe	Burseraceae	Tree
*Bursera aptera* Ramírez	Cuajiote amarillo	Burseraceae	Tree
*Bursera bipinnata* (Moc. & Sessé ex DC.) Engl	Copal chino	Burseraceae	Tree
*Bursera copallifera* (Sessé & Moc. ex DC.) Bullock	Copal	Burseraceae	Tree
*Echinocactus platyacanthus* Link & Otto	Biznaga	Cactaceae	Succulent
*Opuntia streptacantha* Lem	Nopal	Cactaceae	Succulent
*Stenocereus griseus* (Haw.) Buxb	Pitayo	Cactaceae	Succulent
*Stenocereus stellatus* (Pfeiff.) Riccob	Pitayo	Cactaceae	Succulent
*Celtis caudata* Planch	Palo sosonaco	Cannabaceae	Tree
*Ipomoea wolcottiana* Rose	Cazahuate	Convolvulaceae	Tree
*Acacia cochliacantha* Willd	Cubata negra	Fabaceae	Shrub
*Acacia coulteri* Benth	Palo blanco	Fabaceae	Tree
*Acacia farnesiana* (L.) Willd	Huizache	Fabaceae	Shrub
*Acacia pennatula* (Schltdl. & Cham.) Benth	Cubata blanca	Fabaceae	Shrub
*Conzattia multiflora* (Robinson) Standl	Palo totole	Fabaceae	Tree
*Lonchocarpus caudatus* Pittier	Tapachichi	Fabaceae	Tree
*Lysiloma divaricatum* (Jacq.) J.F.Macbr	Tlahuitolo	Fabaceae	Tree
*Mimosa benthamii* J.F.Macbr	Tecolhuixtle	Fabaceae	Shrub
*Piptadenia viridiflora* (Kunth) Benth	Huamuchilillo	Fabaceae	Tree
*Pithecellobium acatlense* Benth	Barba de chivo	Fabaceae	Shrub
*Styphnolobium burseroides* M. Sousa, Rudd & González Medrano, Francisco	Pico de pájaro	Fabaceae	Shrub
*Cassia emarginata L*	Vara de san José	Fabaceae	Shrub
*Bunchosia lanceolata* Turcz	Nanche de zorro	Malpighiaceae	Shrub
*Heliocarpus terebinthinaceus* (DC.) Hochr	Calahuate	Tiliaceae	Shrub

Fabaceae had the highest number of species with 12, while seven other families were represented by one species each. The Fabaceae family also has the highest number of genera, with nine. The Cactaceae and Burseraceae families, which contain species such as *Bursera bipinnata* and *Bursera copallifera*, respectively, from which copal resin is extracted, were the second most species-rich families in the study area (represented by four species).

### Importance value index

#### Relative abundance

Relative abundance results indicate that *Opuntia streptacantha* had the highest abundance in the study area, followed by *Echinocactus platyacanthus* and *Acacia pennatula*. The species with the lowest relative abundance were *Bursera aloexylon, Bursera aptera, Conzattia multiflora, Cyrtocarpa procera, Lysiloma divaricata, Agave karwinskii, Heliocarpus terebinthinaceus, Celtis caudata, Styphnolobium burseroides, Bursera copallifera, Cassia emarginata* and *Marsdenia zimapanica*, each with a relative abundance of less than 1%. *Bursera bipinnata* and *Bursera copallifera*, which are used to produce copal resin, were relatively rare species, with relative abundances of 1.9% and 0.8% respectively, when compared to *Opuntia streptacantha*, which was the species with the highest abundances in the study site.

#### Relative frequency

Relative frequency results reveal that, similar to relative abundance results, *Opuntia streptacantha* had the highest frequency in the study site, followed by *Ipomoea wolcottiana*, and *Acacia pennatula*. The species with the lowest relative frequency were *Agave karwinskii*, *Bursera aloexylon, Bursera aptera, Cassia emarginata, Conzattia multiflora, Cyrtocarpa procera, Heliocarpus terebinthinaceus* and *Lysiloma divaracata*. *Bursera bipinnata* had a relative frequency of 5% and *Bursera copallifera* had a relative frequency of 2.3% (Table 2).

**Table 2:** The relative abundance, frequency and dominance of species, and Importance Value Index (IVI) of species present in the Ejido de San Juan Raboso, Izúcar de Matamoros, Puebla, (arranged in descending order by value of their IVI).

Scientific name	Relative abundance		Relative frequency		Relative dominance		IVI
	ind. ha^−1^	%	Sites	%	m^2^ ha^−1^	%	%
*Opuntia streptacantha*	609	34.0	49	14.3	119.6	2.6	17.0
*Ipomoea wolcottiana*	74	4.1	30	8.7	920.3	20.2	11.0
*Ceiba aesculifolia*	26	1.4	12	3.5	808.2	17.7	7.6
*Acacia pennatula*	113	6.3	29	8.5	227.1	5.0	6.6
*Bursera bipinnata*	33	1.9	17	5.0	440.1	9.7	5.5
*Lonchocarpus caudatus*	94	5.3	11	3.2	327.1	7.2	5.2
*Stenocereus griseus*	96	5.4	24	7.0	108.9	2.4	4.9
*Stenocereus stellatus*	104	5.8	27	7.9	39.9	0.9	4.8
*Acacia farnesiana*	69	3.8	24	7.0	155.5	3.4	4.7
*Echinocactus platyacanthus*	148	8.3	18	5.2	18.6	0.4	4.6
*Mimosa benthami*	70	3.9	11	3.2	159.7	3.5	3.5
*Piptadenia viridiflora*	46	2.6	13	3.8	192.4	4.2	3.5
*Acacia coulteri*	30	1.7	14	4.1	209.4	4.6	3.4
*Bunchosia lanceolata*	91	5.1	12	3.5	25.6	0.6	3.0
*Acacia cochiliacantha*	59	3.3	12	3.5	67.0	1.5	2.8
*Bursera copallifera*	15	0.8	8	2.3	205.3	4.5	2.6
*Styphnolobium burseroides*	9	0.5	5	1.5	147.3	3.2	1.7
*Pithecellobium acatlense*	24	1.3	8	2.3	42.5	0.9	1.5
*Agave ghiesbreghtii*	26	1.4	5	1.5	20.4	0.4	1.1
*Cyrtocarpa procera*	2	0.1	1	0.3	105.1	2.3	0.9
*Masdenia zimapanica*	17	0.9	4	1.2	10.6	0.2	0.8
*Celtis cauadata*	6	0.3	2	0.6	47.5	1.0	0.6
*Conzattia multiflora*	2	0.1	1	0.3	46.0	1.0	0.5
*Cassia emarginata*	17	0.9	1	0.3	2.1	0.05	0.4
*Bursera aptera*	2	0.1	1	0.3	36.4	0.8	0.4
*Lysiloma divaracata*	2	0.1	1	0.3	29.4	0.6	0.3
*Bursera aloexylon*	2	0.1	1	0.3	23.3	0.5	0.3
*Heliocarpus terebinthinaceus*	4	0.2	1	0.3	18.2	0.4	0.3
*Agave karwinskii*	4	0.2	1	0.3	4.2	0.1	0.2
	1793	100.00	343	100.00	4557.8	100.00	100.00

#### Relative dominance

Based on the relative dominance results, the most dominant species were mainly arboreal. In the study area, *Ipomoea wolcottiana* had the highest tree coverage, followed by *Ceiba aesculifolia*, and *Bursera bipinnata*. *Longchocarpus caudatus* was the shrub species with the highest percentage of cover. *Cassia emarginata, Agave karwinskii, Marsdenia zimapanica, Agave ghiesbreghtii, Echinocactus platyacanthus, Heliocarpus terebinthinaceus, Bursera aloexylon, Bunchosia lanceolata, Lysiloma divaracata, Bursera aptera, Pithecellobium acatlense* and *Stenocereus stellatus* were the species with less than 1% canopy cover (Table 2).

#### Importance value index, IVI

In the study area, the Fabaceae family had the highest importance value index, followed by the Cactaceae family. *Opuntia streptacantha* was the species with the highest IVI, followed by *Ipomoea wolcottiana*, and *Ceiba aesculifolia*. *Agave karwinskii, Heliocarpus terebinthinaceus, Bursera aloexylon, Lysiloma divaracata, Bursera aptera* and *Cassia emrginata* were the species with the lowest ecological weight within the study area, all with an IVI of less than 0.5%. The Burseraceae family achieved an IVI of 8.8%, and *Bursera bipinnata* was the best-represented species, with 5.5%, as opposed to *Bursera copallifera*, with 2.6% (Table 2).

### Measure of species diversity

Species diversity in a plant community can be defined in terms of a set of species, each of which has an abundance value that characterizes it^40^.

**Figure 3:**
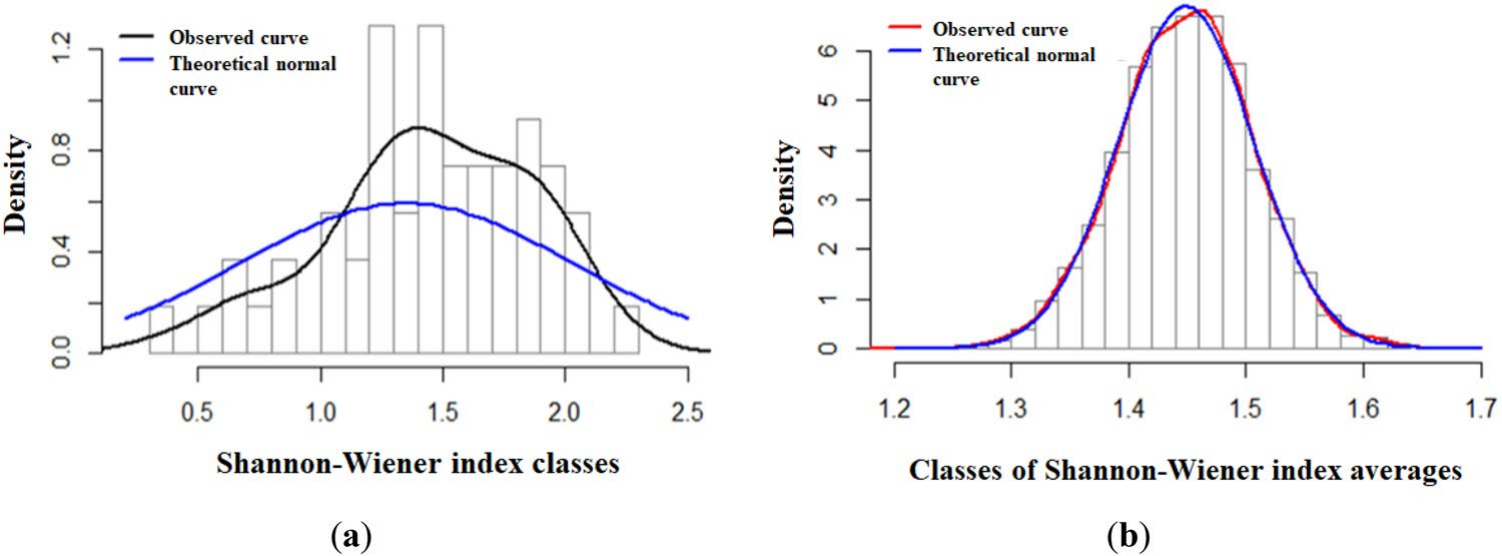
Observed and Bootstrapped distributions of $H$: (**a**) Observed distribution of $H$; (**b**) Bootstrap distribution of ${\overline{H} }^{*}$.

Shannon index. Based upon 54 sampling units, the average Shannon index was ${H}_{average}= 1.44759$, with a standard deviation of ${H}_{sd}= 0.422139$ and a maximum and minimum of ${H}_{Maximum} = 2.243153$ and ${H}_{Minimum} = 0.3488321$, respectively. Its empirical distribution showed a slight negative skewness. Figure 3 presents a histogram of the observed values compared with the expected normal distribution. The goodness-of-fit test (Lilliefors) confirmed that the data corresponding to the random sample were normal $(p-value = 0.7898<0.05)$. The estimated mean for the bootstrap parameter was ${\overline{H} }^{*}=1.44813$, with a bootstrap distribution standard deviation of ${\sigma }_{H}^{*}=0.05784422$ and a bias of 0.00054 $\left[abs\left({H}_{average}= 1.44759-{\overline{H} }^{*}=1.44813\right)\right]$. The 95% confidence interval for this statistic was $\left(1.332< {\overline{H} }^{*}<1.556\right)$ with a width of 0.224.

**Figure 4:**
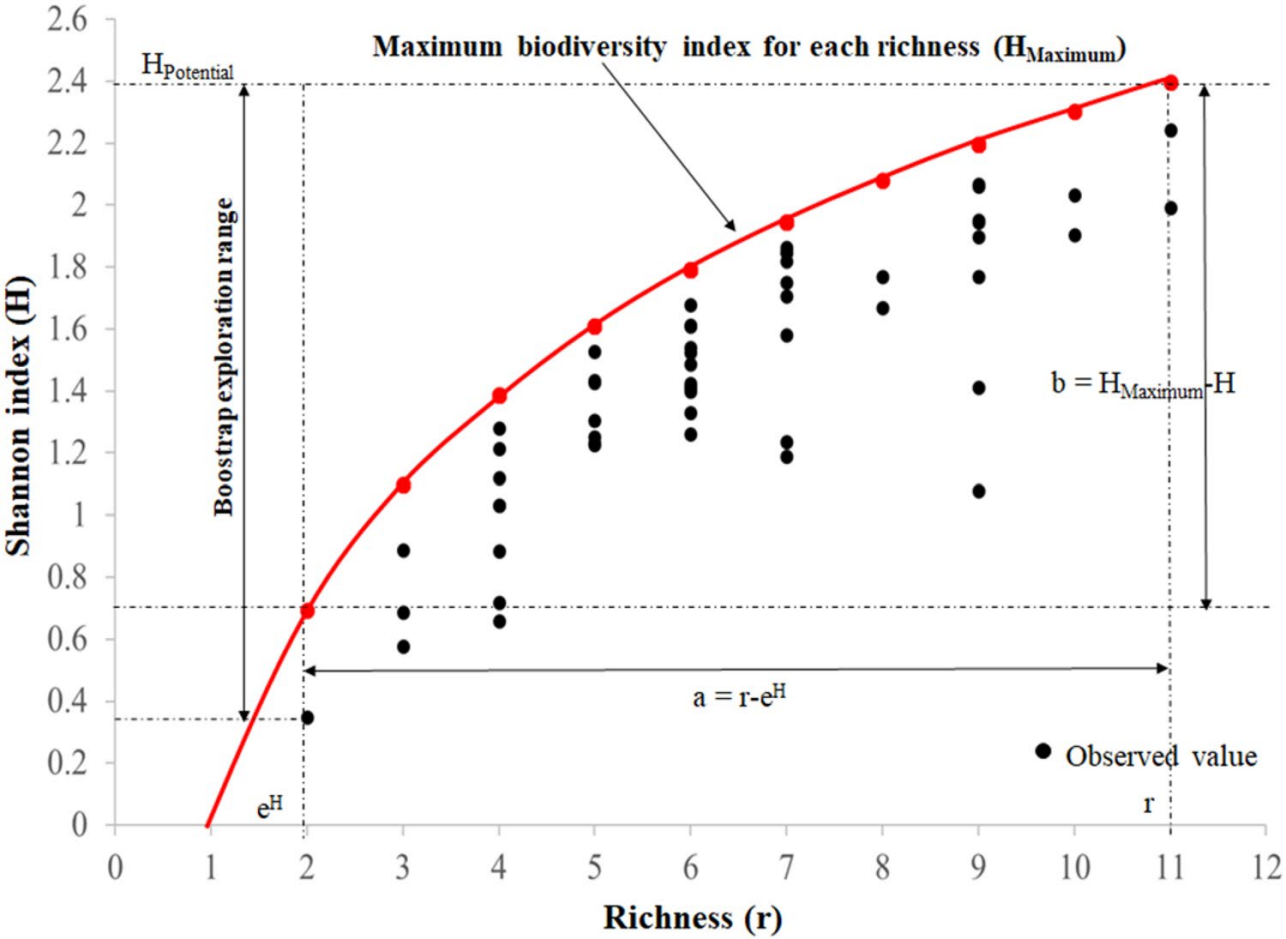
Shannon index as a function of richness. $a={r-e}^{H}$, difference between observed and equiabundant richness; ${H}_{Maximum}-H$, difference between potential and observed Shannon index.

Figure 4 shows the potential Shannon index with the sample data when all species are equally abundant $({H}_{Maximum}=\mathrm{ln}r)$. The ${r}_{H}={e}^{H}$ value indicates how many species with equal abundance would be required to obtain the observed index. Horizontal deviations ($a={r-e}^{H}$: differences between observed and potential species richness when all species are equally abundant) indicate the number of species present in the community that have not reached their maximum equity, which refers to all species having the same number of individuals, or the same abundance^27^ with respect to the number of species needed for the observed Shannon index to be maximal. The greater the difference between ${e}^{H}$ and $r$, the total species, the fewer species there will be in the community, and the less diverse the community will be. Vertical deviations ($b={H}_{Maximum}-H$: difference between the potential and observed Shannon indexes) indicate how much of the potential diversity did not manifest itself in the observed community when all species are equally abundant. The richness axis was defined as ${e}^{H=0}$=1 since every community will contain at least one species with a Shannon index of 0, i.e. $H=0$. ${H}_{average}=1.44759$, ${H}_{Maximum}=2.243153$ and ${H}_{Minimum}=0.3488321$. This community is considered to have a slightly average level of diversity.

**Figure 5:**
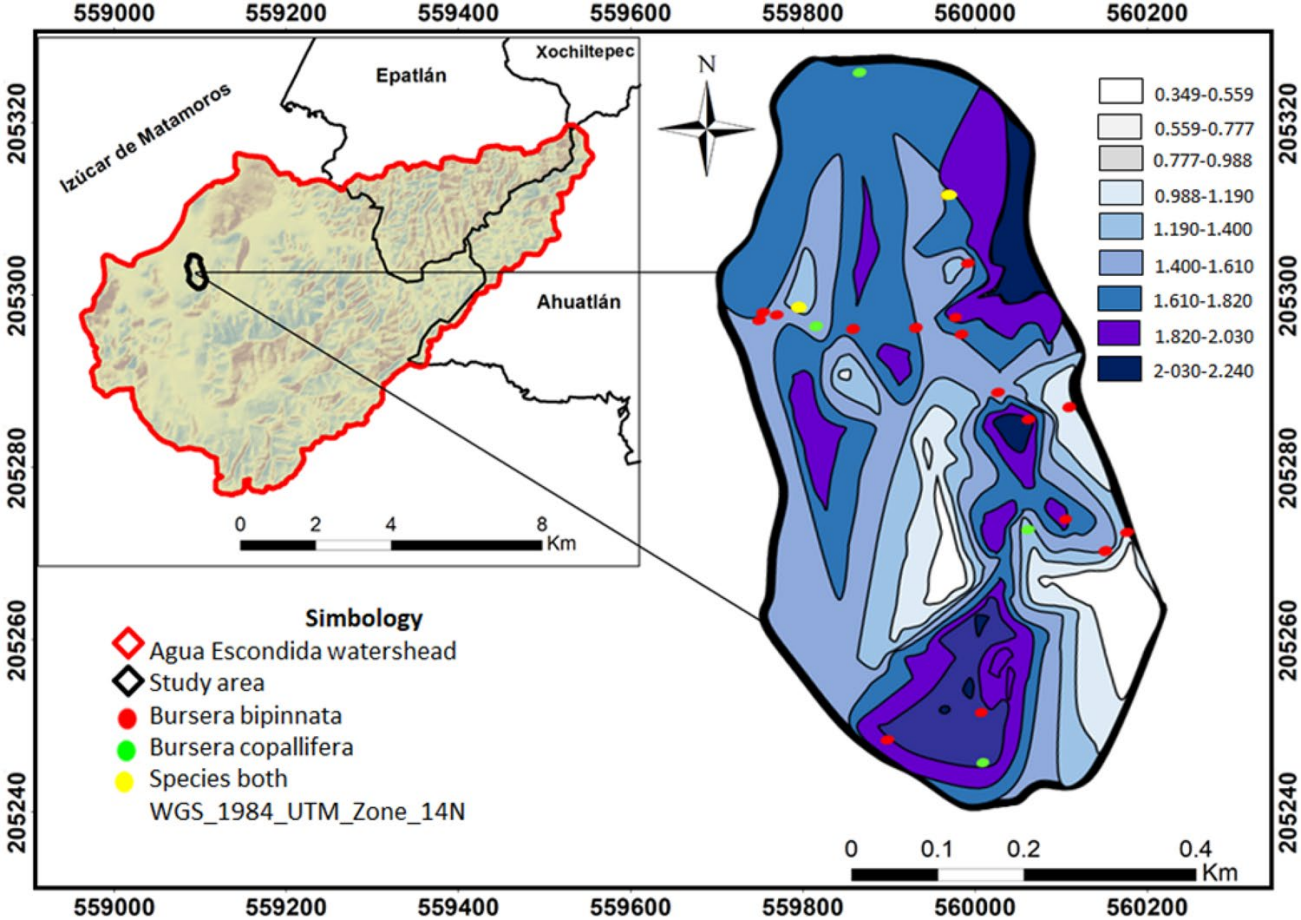
Interpolation of the Shannon index of *Bursera bipinnata* and *Bursera copallifera* species in 54 sample units (Elaborated with ArcGIS 10.8, https://pro.arcgis.com/es/pro-app/latest/get-started/download-arcgis-pro.htm and R Project, https://www.r-project.org/).

Figure 5 shows the values obtained for the Shannon index and richness in the study area. Light colored areas indicate sites with low Shannon values $({H}_{Minimum}= 0.3488321$), i.e. sites with low diversity, while colors with dark shading indicate sites with medium Shannon values $({H}_{Maximum}=2.243153)$, i.e. sites with medium diversity. Figures 5 also show the distribution of *Bursera bipinnata* and *Bursera copallifera* in the study area in red and green, respectively, and in orange when both species are present. The presence of these species is concentrated in areas with Shannon indices ranging from 1.19 to 2.40, which indicates that they are found in areas with medium diversity according to the classification proposed by Aguirre^31^. In spite of this, most of the copal species are found on sites with a diversity of about 1.40–1.610, which is considered to be a slightly medium diversity of sites.

## Discussion

### Floristic composition

Based on this study, the family with the greatest species richness was Fabaceae, which is consistent with research carried out in tropical deciduous forests within warm climate zones^8,41^. The Burseraceae and Cactaceae families were represented by four species, as in the study conducted in the tropical deciduous forest in the Balsas region of Michoacán, where seven and four species, respectively, were found in areas of cattle ranching and agriculture^42^. Both *Bursera* and *Acacia* were the genera with the largest number of species within the Ejido de San Juan Raboso, with four species each. The former is of particular interest for its endemism and diversity, being native to the Balsas watershed, one of the major extensions of the dry forest^43^, whereas the latter is a genus with an extensive distribution and a preference for tropical deciduous forests^8^.

### Importance value index

#### Relative abundance

The species *Opuntia streptacantha* and *Echinocactus platyacanthus*, both of which belong to the family Cactaceaea, were the most abundant in the Ejido San Juan Raboso. Families of this genus belong to the physiognomy of tropical deciduous forests^44^ because they have morphological and physiological traits that have allowed them to emerge in both arid and warm environments^45^. *Acacia pennatula* occupied an important position in terms of species abundance in the study area because it is one of the most diverse families within this vegetation type^46^. *Bursera bipinnata* and *Bursera copallifera*, two species from which copal is extracted, are typical of tropical deciduous forests; however, their abundance was not representative when compared with *Opuntia streptacantha* and *Echinocactus platyacanthus.* This is primarily due to both ecological and anthropogenic factors. The former is determined by climatic variables that determine its distribution, whereas the latter is determined by the amount of exploitation by residents of the community on natural copal populations^47^, mainly due to the lack of knowledge of the proper technique for resin extraction, in addition to the fact that extraction times and rest periods for each individual are not respected^12^.

#### Relative frequency

*Opuntia streptacantha, Ipomoea wolcottiana, Acacia pennatula* and *Stenocereus stellatus* were the species with the highest frequency. This is due to the fact that its morphological and physiological characteristics are typical of tropical deciduous forests^44^. *Ipomoea wolcottiana*'s relative frequency coincides with that reported by Alcántar-Mejía et al.^48^, who discovered that this genus is more frequent in tropical deciduous forests than other types of vegetation. Similarly, Rojas-Martínez and Flores-Olvera^46^ also identify *Acacia pennatula* as one of the characteristic species of tropical deciduous forests.

#### Relative dominance

Results of this study similar to those of Alcántar-Meja et al.^48^, who found that species of *Ipomoea* prefer dry warm environments; as a consequence, it is important to emphasize the role of trees in tropical deciduous rainforests, as well as the contribution of other life forms including shrubs and cacti in this type of habitat^43^. There were low species richness in more than 50% of the study area, ranging from two to five species. *Bursera bipinnata* and *Bursera copallifera* are two of them, which probably indicates overexploitation, changes in the community structure and lack of species regeneration of species^44^.

#### Importance value index

According to the results of this study, *Opuntia streptacantha, Ipomoea wolcottiana,* and *Ceiba aesculifolia* were the species with the greatest ecological weight in the study area; however, these results differ from the results of Alanís et al.^49^ and Hernández et al.^50^ in the tropical deciduous forests of San Luis Potosí and Morelos, Mexico. Alanís et al.^49^ concluded that *Lysiloma* had the highest ecological weight and *Lysiloma divaricata* had the highest IVI, with 39.98%, which is a consequence of anthropogenic use in the area as well as the fact that local producers use trees from this genus as living fences. Hernández et al.^50^ found that *Lysiloma divaricata* was also one of the most ecologically significant species in the tropical deciduous forests of three environmental management units in the Sierra de Huautla, Morelos, because of its abundance, anthropogenic importance, and value as a wildlife food. Both the *Bursera bipinnata* and *Bursera copallifera* species demonstrated low IVI values (5.5% and 2.6%, respectively), as opposed to what was reported by Sánchez et al.^51^, in which *B. copallifera* is one of the species that exhibited the highest IVI values, with 18.03%, in the tropical deciduous forest of San Luis Potosí, Mexico. This may be due to the low abundance and frequency of species in the sampling area as a result of overexploitation of species for copal extraction, which may alter the structure and development of this vegetation type^44,47^.

### Measurement of species diversity

Species richness manifests various aspects of biodiversity^25^. The Shannon index is one of the most widely used measures of biodiversity, based on the number of species and their relative abundance^29^. ${H}^{^{\prime}}$ index increases with an increase in the number of species and when individuals are more homogeneously distributed among all species present^52^. Shannon's Index indicates that maximum equity, which refers to all species having the same number of individuals, or the same abundance^29^, did not exist in the study area since some of the species were more abundant than others, indicating a change in the ecosystem's structure mainly caused by anthropogenic activity^31^. According to the classification proposed by Aguirre^31^, the average Shannon index value (${H}_{average}=1.44759$) indicated that the study area was moderately diverse. Among the possible reasons for the difference between the average Shannon index value and the maximum potential value could be the intensive use of copal resin to generate economic income^12^. Similar results were found in the study developed in tropical deciduous forests in Sierra de Huautla Biosphere, Morelos, Mexico, which obtained a mean value of the Shannon index of 1.6. This region is the most diverse in the state, but despite being a protected area, it doesn't ensure the conservation of biodiversity^51^. In the tropical deciduous forest of Tziritzícuaro Balsas depression in Michoacán, Mexico, a medium diversity of species was also reported^8^. However, these results differ from those reported by Alanís et al.^49^ in the tropical deciduous forest in San Luis Potosí, where a high diversity was documented, a difference that might be explained by the fact that the capture sites are in areas without indications of disturbance. In the study area, in addition to the intensity of copal resin harvesting, overgrazing is a key factor in the decline of the species, mainly due to sheep, since this type of livestock enters the tropical deciduous forest areas, affecting the renewal of the species^15^. Likewise, the impact of the local population collecting these species for use as firewood should not be overlooked, since firewood is a unique or complementary energy source for families, and outstanding element in the decrease in diversity^53^. In general, ecosystems are comprised of a number of components, all of which are essential for their proper functioning. When one of these components is lost, they may cease to function properly. Therefore, the Shannon index is an important indicator of species diversity, since a loss of biodiversity can degrade the functionality of an ecosystem, and can even lead to a collapse in severe circumstances. As a result, the absence of a system component is often much more critical to determining whether a population will persist or disappear^54,55^.

## Conclusions

The deciduous tropical forest of Alto Balsa Poblano contains a variety of plants that represent valuable natural capital, in particular, species of the Bursera genus, from which the aromatic resin, called copal, is extracted. The Fabaceae family presented the highest number of species followed by the Cactaceae and the Burseraceae. However, the diversity values were moderate due to the low richness of species present and the different abundances, so maximum equity was not achieved. This may be due to anthropic activities such as the intensive extraction of copal resin, without rest time for the resinated trees, and lack of technical specifications in the extraction method, as well as the constant overgrazing in the area, extraction of succulent plants and no activities of complementary management, which could affect the alteration of the structure of this ecosystem. The continuation of these trends could threaten the existence of many keystone species and, as a result, the viability of the ecological systems on which the local economy depends. Although the great importance of this type of vegetation is well known, it is in an accelerated process of deterioration of its natural resources. Therefore, the conservation of biodiversity is essential for the survival of endemic species, so it is necessary to address the social and cultural factors that currently motivate the irrational use of these resources. Studies of this type can help us understand the current state of plant communities and serve as a basis for future research in order to propose strategies to conserve natural forests.

## Data Availability

The analyzed datasets are available from the corresponding author on reasonable request.
